# Custom UAV with model predictive control for autonomous static and dynamic trajectory tracking in agricultural fields

**DOI:** 10.3389/frobt.2025.1694952

**Published:** 2025-12-16

**Authors:** Veera Venkata Ram Murali Krishna Rao Muvva, Kunjan Theodore Joseph, Yogesh Chawla, Santosh Pitla, Marilyn Wolf

**Affiliations:** 1 Department of Biological Systems Engineering, School of Computing, University of Nebraska–Lincoln, Lincoln, NE, United States; 2 Department of Biological Systems Engineering, University of Nebraska–Lincoln, Lincoln, NE, United States; 3 School of Computing, University of Nebraska–Lincoln, Lincoln, NE, United States

**Keywords:** autonomous UAV, model predictive control, Kalman filter, trajectory tracking, drones

## Abstract

**Introduction:**

This study introduces a custom-built uncrewed aerial vehicle (UAV) designed for precision agriculture, emphasizing modularity, adaptability, and affordability. Unlike commercial UAVs restricted by proprietary systems, this platform offers full customization and advanced autonomy capabilities.

**Methods:**

The UAV integrates a Cube Blue flight controller for low-level control with a Raspberry Pi 4 companion computer that runs a Model Predictive Control (MPC) algorithm for high-level trajectory optimization. Instead of conventional PID controllers, this work adopts an optimal control strategy using MPC. The system also incorporates Kalman filtering to enable adaptive mission planning and real-time coordination with a moving uncrewed ground vehicle (UGV). Testing was performed in both simulation and outdoor field environments, covering static and dynamic waypoint tracking as well as complex trajectories.

**Results:**

The UAV performed figure-eight, curved, and wind-disturbed trajectories with root mean square error values consistently between 8 and 20 cm during autonomous operations, with slightly higher errors in more complex trajectories. The system successfully followed a moving UGV along nonlinear, curved paths.

**Discussion:**

These results demonstrate that the proposed UAV platform is capable of precise autonomous navigation and real-time coordination, confirming its suitability for real-world agricultural applications and offering a flexible alternative to commercial UAV systems.

## 1 Introduction

Uncrewed Aerial Vehicles (UAVs) (Austin, 2011; Gundlach, 2014; Garg, 2021) have become transformative tools in agriculture, reshaping traditional practices through enhanced data collection, targeted input application, and improved efficiency. As agriculture shifts toward data-driven decision-making, UAVs play a critical role in remote sensing by providing high-resolution spatial and temporal data for assessing crop health, field variability, and resource management (Zhang and Zhu, 2023). Their ability to rapidly survey large areas enables near-real-time monitoring of vegetation indices, soil conditions, and plant development stages.

Within precision agriculture, UAVs facilitate site-specific management by enabling the precise delivery of water, fertilizers, and agrochemicals. This targeted approach minimizes resource waste, reduces environmental impact, and improves crop productivity. UAVs are also used extensively for crop scouting, plant classification, and monitoring of agronomic indicators such as biomass, canopy cover, and chlorophyll levels (Bendig et al., 2014; Kalischuk et al., 2019). When equipped with multispectral, thermal, or hyperspectral sensors, UAVs can detect early signs of water stress, pest infestations, weed intrusion, and plant diseases (Zhang et al., 2019; Gašparović et al., 2020), enabling timely interventions that reduce potential yield loss. The integration of advanced technologies, such as machine learning, deep learning (Muvva et al., 2021), and the Internet of Things (IoT) has further expanded UAV capabilities by enabling adaptive mission planning, automated decision-making, and seamless connection with farm management systems (Rejeb et al., 2022). Recent work even demonstrates autonomous landing on static and moving platforms (Muvva et al., 2022), underscoring the potential of cooperative aerial–ground systems.

Beyond the functionality of individual aerial platforms, the integration of UAVs with Uncrewed Ground Vehicles (UGVs) establishes a cooperative system that exploits the complementary capabilities of both agents. While UAVs deliver rapid, large-scale sensing and can also support repetitive tasks such as payload refilling or relay operations more efficiently, UGVs perform high-precision tasks on the ground, including targeted spraying, soil sampling, and mechanical weeding. Extensive research has focused on developing frameworks and coordination strategies for such UAV–UGV collaboration. Ou et al. (2024) developed a decentralized UAV–UGV collaboration framework that integrates an information consensus filtering approach with CBF–CLF control principles, enhancing cooperative localization accuracy and operational safety. Rao et al. (2025) presented a cooperative localization strategy that fuses deep learning–based object detection with Kalman filtering, achieving sub-meter positioning accuracy for UAV–UGV teams even in conditions where GNSS performance is limited. However, realizing the full potential of such UAV–UGV cooperative systems requires flexible and research-oriented aerial platforms with capabilities often constrained in commercial solutions.

Operating UAVs in agricultural fields is difficult due to strong winds, uneven terrain, and crop canopy effects that affect stable flight. This demands adaptive controllers that respond to disturbances instead of relying on fixed gains. Simulation is crucial for safe, repeatable testing before deployment. For example, Ahmed and Xiong (2024) proposed an adaptive impedance control method in a Unity-based simulator (CoppeliaSim), adjusting parameters like stiffness in real time to improve disturbance rejection. Their tests across various trajectories and motion speeds showed better performance than PID and MRAC controllers. Similarly, Goldschmid and Ahmad (2025) demonstrates in simulation that a quadratic MPC significantly outperforms traditional PID-based controllers through step-response, circular, figure-eight, and obstacle-avoidance trajectory experiments on the Parrot Anafi platform, achieving lower tracking errors and smoother control performance. However, simulation alone is not enough. Field experiments are needed to capture real environmental effects and validate system performance. Bridging the gap between controlled simulation and complex field conditions often requires hardware-level customization and flexible platforms, which commercial UAVs struggle to provide.

Despite these advances, commercial UAVs (e.g., DJI Inspire 2, Phantom series, Matrice platforms) present limitations for research and specialized applications (Seidaliyeva et al., 2024). While they offer robustness and ease of operation, their proprietary software and hardware ecosystems restrict low-level control access (Mohsan et al., 2023), payload customization, and algorithmic flexibility capabilities essential for academic research and development of novel autonomy frameworks (Yang et al., 2023). Furthermore, the rapid evolution of commercial UAV ecosystems with frequent hardware revisions, API changes, and discontinued support—creates challenges for long-term research and system integration (Nex et al., 2022). Oguz et al. (2024) developed a streamlined, open-source UAV framework for swarm robotics research, demonstrating effective performance in controlled indoor environments. Their work, while valuable for establishing a reproducible experimental baseline, remains limited to laboratory conditions with constrained environmental variability. In contrast, our research extends this framework to real-world outdoor scenarios, addressing the increased challenges of environmental uncertainty, wind disturbances, and larger operational areas inherent to field testing.

Custom UAV development, in contrast, offers an open and adaptable framework that allows researchers to select flight controllers, sensors, computing modules, and airframes tailored to specific mission needs and budgets. It also enables rapid prototyping and iterative testing of novel control algorithms, perception systems, and navigation architectures. The use of open-source autopilot software such as ArduPilot (Peksa and Mamchur, 2024) further enhances the value of custom platforms by supporting advanced flight modes, parameter tuning, and the integration of experimental strategies such as Model Predictive Control (MPC) or reinforcement learning.

Developing a custom UAV, however, introduces engineering challenges in areas such as component selection, integration, power management, and software configuration (Bhat et al., 2024). These challenges require interdisciplinary expertise spanning mechanical design, electronics, and control systems. Nevertheless, many of these barriers can be mitigated through modular design principles and the adoption of community-supported hardware. Strategic planning allows researchers to streamline the development cycle, reducing integration effort and enabling focus on experimentation.

Another important consideration is regulatory compliance. UAV standards vary across regions, influencing design choices related to flight altitudes, weight limits, communication protocols, and operational safety. For example, UAVs used near urban areas or mountainous terrain may require different sensing and communication strategies compared to those operating in open farmland. Custom development provides the flexibility to adapt systems to local regulations while incorporating mission-specific features (Muthukumar, 2023).

Given these factors, this research presents the design of a custom UAV platform with modular communication, onboard intelligence, and support for advanced control strategies such as Model Predictive Control (MPC). The system accommodates both off-the-shelf and custom sensors, balancing adaptability, scalability, and cost-effectiveness. Although custom UAV development demands greater initial effort, the resulting platform offers resilience, flexibility, and research-oriented functionality, making it a compelling alternative where commercial UAVs fall short. To address this, the present work implements a MPC framework designed for real-time trajectory generation and tracking. This controller enables the UAV to proactively plan its motion, intercept the UGV’s route, and maintain a hovering position relative to the ground vehicle throughout its operation (Budiyanto et al., 2015; Falanga et al., 2019; 2020).

Validation of the system was carried out through both simulated and real-world testing. A software-in-the-loop (SITL) environment was constructed using AirSim (Shah et al., 2018), ArduPilot (Audronis, 2017), and ROS (Quigley et al., 2015), allowing for algorithm verification in a controlled, high-fidelity simulation setup prior to deployment. Furthermore, a physical test platform was built using the CubePilot flight controller, facilitating extensive outdoor testing in a 300-acre agricultural field. The UAV successfully tracked the UGV in real-time, demonstrating the applicability and robustness of the proposed solution.

The principal contributions of this research are summarized as follows:.

**FIGURE 1:**
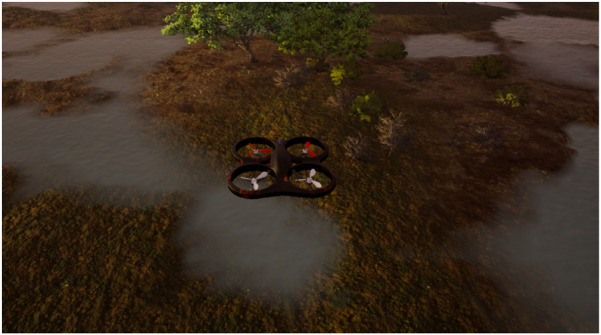
A scene from simulator.

**FIGURE 2:**
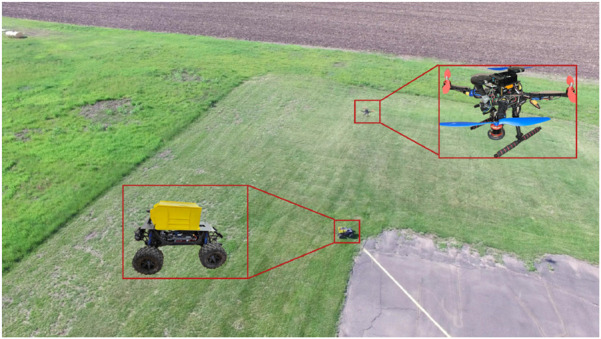
A scene from field test.

- Development of a Fully Autonomous UAV Platform–A custom UAV was built from the ground up, integrating both the physical hardware and on-board software stack for autonomy.
- MPC-Based Trajectory Tracking–Implementation of a Model Predictive Controller for generating smooth, real-time control inputs to follow predefined or dynamically changing trajectories.
- UAV–UGV Coordination Using Sensor Fusion–A cooperative localization and control framework was developed, integrating Kalman Filtering with MPC to enable aerial-ground vehicle tracking.
- Field-Level Experimental Verification–Real-world deployment and testing of the UAV-UGV system in a large agricultural setting to establish its operational reliability and accuracy (refer Figures 1, 2).

## 2 Materials

### 2.1 Physical plant

#### 2.1.1 Flight controller

**FIGURE 3:**
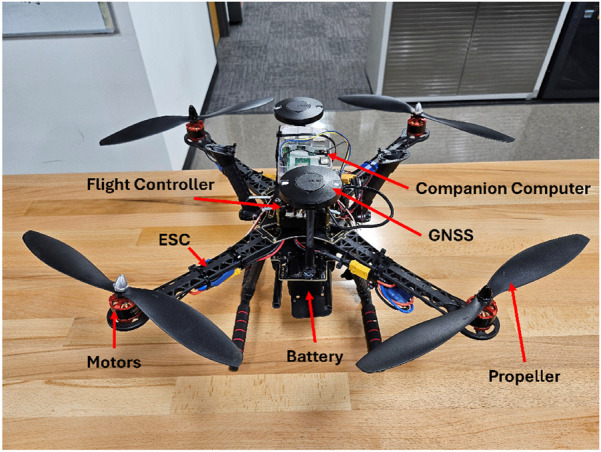
Components of custom UAV.

The Cube Blue flight controller (Cube-Blue-FC, 2025) functions as the primary control unit of the UAV, chosen for its modular architecture, advanced performance, and reliability across diverse uncrewed aerial applications. As part of the CubePilot ecosystem, it is designed for seamless integration with a wide range of sensors and peripherals, allowing flexible configuration to meet different mission objectives. One of its defining features is the inclusion of triple redundant Inertial Measurement Units (IMUs) and dual barometers. This redundancy improves fault tolerance and ensures accurate state estimation, which is essential for stable flight under variable environmental conditions. The controller also supports multiple communication interfaces, such as CAN and I2C, facilitating efficient connectivity with GNSS receivers, telemetry links, and other onboard modules. With high-speed processing and robust sensor fusion capabilities, the Cube Blue provides precise real-time navigation and control. Its rugged build and adaptability make it particularly suited for complex UAV missions, including precision agriculture, where dependable performance and rapid decision-making are critical. The main hardware components of the custom UAV and their arrangement are shown in Figure 3.

#### 2.1.2 Sensors

##### 2.1.2.1 IMU

The Cube Blue flight controller integrates three redundant Inertial Measurement Units (IMUs), which are essential for estimating the UAV’s orientation, angular velocity, and linear acceleration. Each IMU contains a 3-axis gyroscope and a 3-axis accelerometer, providing six degrees of motion sensing. The redundancy improves fault tolerance by allowing the system to switch to alternate IMUs if one unit fails or delivers inconsistent readings. This multi-IMU setup significantly enhances reliability and stability, especially during aggressive maneuvers or in environments with vibration and potential sensor drift.

##### 2.1.2.2 Barometer

Alongside the IMUs, the Cube Blue incorporates dual barometric pressure sensors for altitude estimation. These sensors measure atmospheric pressure to infer relative altitude, which is critical for stable takeoff, landing, and maintaining designated flight levels. The dual-barometer setup allows cross-checking of measurements, minimizing errors caused by sensor faults or environmental variations such as pressure fluctuations. This redundancy is especially valuable in low-altitude missions, where precise vertical control is required for applications like precision agriculture and close-proximity inspection.

##### 2.1.2.3 GNSS

This study employed the HereLink GNSS module (Here3-GNSS, 2025) to deliver accurate and reliable positioning for the UAV. The receiver supports multiple satellite constellations, including GPS (United States), GLONASS (Russia), Galileo (EU), and BeiDou (China), collectively referred to as GNSS. Leveraging signals from several constellations simultaneously improves satellite visibility, shortens the time to first fix (TTFF), and increases overall positioning precision. Such multi-constellation capability is particularly advantageous in agricultural fields, where signals may be obstructed by trees, structures, or uneven terrain. Under optimal conditions and with correction services, the HereLink GNSS achieves centimeter-level accuracy, making it well-suited for high-precision UAV applications. Its rugged design ensures consistent performance across varying environmental conditions, including temperature shifts, wind, and dust exposure. This level of reliability is essential for autonomous missions requiring precise waypoint tracking, systematic area coverage, and repeatable flight paths.

#### 2.1.3 Actuators

##### 2.1.3.1 Motors

The UAV’s propulsion system is powered by four Readytosky 2212 920 KV brushless DC (BLDC) motors, chosen for their balance of thrust, efficiency, and mechanical reliability. With a KV rating of 920, each motor produces approximately 920 revolutions per minute (RPM) per volt, making them well-suited for stable flight in medium-lift quadcopter configurations. Paired with standard 10 × 4.5 propellers, the motors provide a sufficient thrust-to-weight ratio to support autonomous operations with onboard sensing and computing payloads. The 2,212 series motors measure 28 mm in diameter and 24 mm in height (excluding the shaft) and are optimized for operation at a nominal 12 V, consistent with 3S LiPo battery systems. Both clockwise (CW) and counter-clockwise (CCW) variants are included, which counteract torque imbalance and enhance yaw stability. Pre-installed 3.5 mm bullet connectors simplify wiring by eliminating soldering requirements during integration.

##### 2.1.3.2 ESCs

Motor speed regulation and power distribution are managed by QWinOut 30 A brushless electronic speed controllers (ESCs), each paired with a corresponding motor. Designed for 2S–4S LiPo compatibility, these ESCs support a continuous current of 30 A with short-duration bursts up to 40A. An integrated 5 V 3A Battery Elimination Circuit (BEC) supplies regulated power for the flight controller or auxiliary electronics. Pre-installed with SimonK firmware, the ESCs provide rapid throttle response and optimized signal timing tailored to multirotor platforms. This improves motor actuation precision, contributing to smoother dynamics and stable autonomous flight. For integration, each ESC includes 3.5 mm bullet connectors, allowing quick, solder-free connection to both the motors and the power distribution system. Reliability is further enhanced through built-in safety functions, including low-voltage cutoff, overheat protection, and throttle signal loss detection. Users can also adjust parameters such as motor timing, braking mode, startup profile, and cutoff thresholds, enabling fine-tuned performance for specific mission needs. With a lightweight design (25 g per unit), strong current-handling capacity, and straightforward installation, these ESCs are well suited for UAVs deployed in field applications such as precision agriculture and environmental monitoring.

##### 2.1.3.3 Propellers

The UAV employs 10 × 4.5 inch (1,045) two-blade propellers from Readytosky, chosen for their compatibility with 2212-size brushless motors and suitability for medium-class multirotor platforms. Manufactured from durable ABS plastic, the propellers provide an effective balance of strength and lightweight construction. Their aerodynamic profile ensures efficient thrust production with minimal vibration, enhancing overall flight stability and responsiveness. Each set includes both clockwise (CW) and counter-clockwise (CCW) variants, enabling proper pairing with opposing motors and reducing net torque effects during flight. The “1,045” specification denotes a 10-inch diameter and 4.5-inch pitch, a combination well-suited for UAVs with 450–550 mm frames and moderate KV motors (800–1,100 KV). This size-pitch configuration offers a strong balance between lift capacity and efficiency, supporting reliable hovering while still permitting agile maneuvering.

#### 2.1.4 Companion computer

In this study, the Raspberry Pi 4 served as the onboard companion computer, offering a compact and cost-efficient platform for autonomous UAV operations. Powered by a quad-core ARM Cortex-A72 processor and equipped with 8 GB of RAM, it provided sufficient computational capacity for real-time processing, sensor interfacing, and data logging. The board’s connectivity options—including USB 3.0/2.0, Gigabit Ethernet, Bluetooth, and Wi-Fi—enabled seamless integration with onboard peripherals and wireless ground systems. While multimedia outputs such as dual 4K HDMI were not utilized, the device’s processing resources were dedicated to executing autonomy scripts, handling sensor streams, and communicating with the Cube Blue flight controller via MAVLink protocols. Its lightweight form factor, low power requirements, and strong community support made the Raspberry Pi 4 particularly suitable for field-deployable UAVs, ensuring reliable performance in autonomous flight missions.

#### 2.1.5 Other components

##### 2.1.5.1 Chassis

An off-the-shelf quadcopter frame (29 × 18 × 6 cm, 454 g) made of PCB carbon fiber composite was used, offering strength, low weight, and stability. Its central PCB plate provides integrated solder points for seamless connection of the Cube Blue flight controller, PDB, and Raspberry Pi 4 companion computer, enabling autonomous control and real-time optimization. The frame also supports gimbal systems like a 2-axis brushless GoPro mount, allowing high-resolution imaging and FPV for monitoring and precision agriculture applications.

##### 2.1.5.2 Power distribution board

The custom UAV’s power distribution board (65 × 65 mm, 4 mounting holes) manages power delivery with high reliability and flexibility. It supports up to 60 V (14S), 400 A loads, and provides 12 power pad pairs for up to 12 motors, enabling quad, hexa, or octo configurations. Integrated voltage/current sensors enable real-time monitoring, while dual DC-DC converters (5 V/5 A and 12 V/5 A) supply stable power to peripherals. Compatible with SmartAP and other controllers, the PDB ensures robust and adaptable performance for autonomous UAV missions.

##### 2.1.5.3 Battery

To power the custom UAV, we selected an 11.1 V 5200 mAh LiPo battery (50C) that balances high energy density, compact size, and reliable power delivery. Measuring 5.16 × 1.65 × 1.14 inches and weighing 334.7 g, it adds minimal payload while enabling extended flight times for large-area missions. The 50C rating supports high current demands for rapid maneuvers, autonomous control, and sensor payloads. Seamlessly integrated with the UAV’s power system, it ensures stable voltage supply to all components, critical for reliable operation across varied conditions.

### 2.2 Software in the loop

#### 2.2.1 ArduCopter

ArduCopter (Audronis, 2017), part of the open-source ArduPilot ecosystem, functions as the primary flight control firmware in this study. It offers a comprehensive autopilot stack designed for multirotor platforms, supporting both manual and autonomous modes such as stabilized flight, waypoint navigation, and guided missions. Its modular structure enables seamless integration with sensors, GNSS modules, companion computers, and actuators, making it adaptable across diverse UAV configurations.

In this work, ArduCopter is executed on the Cube Blue flight controller, where it handles low-level control loops, sensor fusion, and real-time trajectory execution. Features such as custom control tuning, mission scripting, and telemetry support were leveraged to implement and validate advanced strategies including optimal control. Moreover, its compatibility with MAVLink ensures reliable communication with the Raspberry Pi companion computer and ground control stations, enabling real-time monitoring, data logging, and command updates during both simulation and field trials.

#### 2.2.2 AirSim

AirSim (Shah et al., 2018) is utilized in this research as the primary simulation platform for designing and validating autonomous UAV control strategies under controlled, repeatable conditions. Developed by Microsoft on the Unreal Engine, it offers high-fidelity physics, photorealistic rendering, and configurable sensor models, making it well-suited for aerial robotics studies. The platform supports multiple vehicle types and provides an extensible API that enables integration of custom control algorithms, perception pipelines, and mission frameworks.

For this work, AirSim is configured to simulate open-field environments and variable wind conditions relevant to agricultural UAV operations. The autonomous stack is deployed within these virtual scenarios prior to field experiments, ensuring a consistent software-in-the-loop (SITL) setup by linking AirSim with ROS 2 and the same control framework used on the physical UAV. This approach facilitates early-stage testing, debugging, and parameter optimization while avoiding the risks and logistical overhead of flight trials. Overall, AirSim significantly accelerates the development cycle and supports safe, reliable controller validation.

#### 2.2.3 ROS2

Robot Operating System 2 (ROS 2) (Rico, 2022) serves as the middleware framework for high-level autonomy, data exchange, and modular integration within the UAV platform. Its distributed architecture supports communication among system components, including sensor drivers, perception modules, control algorithms, and logging utilities. Compared to ROS 1, ROS 2 offers enhanced real-time performance, which is particularly beneficial for latency-critical aerial robotics tasks.

In this work, ROS 2 is deployed on the Raspberry Pi companion computer, coordinating data between the control system, GNSS, IMU streams, and simulation environments such as AirSim. Custom ROS 2 nodes manage UAV state publishing, control command execution, and links to external monitoring interfaces. The framework’s scalability and modularity make it well-suited for future extensions, such as integrating additional sensors or enabling multi-robot coordination.

## 3 Methodology

In motion control literature, trajectory tracking refers to the problem where a vehicle is required to follow a reference path that is explicitly parameterized in time, i.e., both the spatial coordinates and their corresponding timing information are predefined. Conversely, path following focuses on converging to and moving along a desired geometric path without any preassigned timing law, allowing the vehicle to adjust its speed autonomously (He et al., 2025).

**FIGURE 4:**
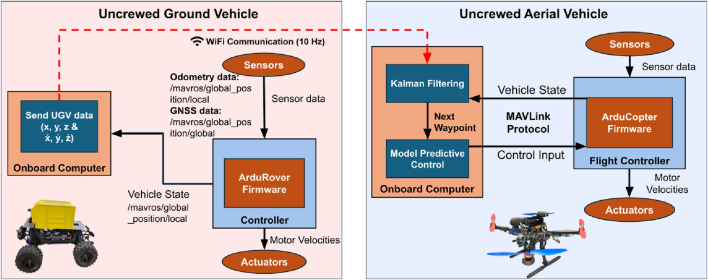
Block diagram of the system.

In our system (Figure 4), these two modes are distributed between agents: the UGV executes a waypoint-based path following task, where it sequentially follows predefined spatial references without explicit timing constraints, while the UAV performs real-time trajectory tracking, generating and tracking a time-varying trajectory based on the live UGV position data. The UAV maintains a hovering offset above the UGV, adapting its motion dynamically as the ground vehicle progresses along its path.

### 3.1 Control

This section details the design and implementation of the optimal control strategy, namely, Model Predictive Control (MPC). It begins with the state-space formulation, specifying the selected system states and inputs and the mathematical model used to represent the UAV dynamics. Building on this representation, the controller derivation is presented, including the definition of the cost function and the finite-horizon optimization procedure used to compute control inputs over the prediction horizon.

UAV state contains linear displacement along front-back (x) , left-right (y) , up-down axis (z) , and angular displacement (ψ) along yaw axis.

$$X={\left[x,\;y,\;z,\;\psi \right]}^{T}$$ (1)

UAV input contains linear velocity along front-back $\left(\dot{x}\right)$ , left-right $\left(\dot{y}\right)$ , up-down $\left(\dot{z}\right)$ axis, and angular velocity $\left(\dot{\psi }\right)$ along yaw axis

$$u=\left[\dot{x},\;\dot{y},\;\dot{z},\;\dot{\psi }\right]$$ (2)

The continuous state space equations can be seen in Equations 3, 4. These equations are shown shorthand in Equation 5

$$\left[\begin{array}{c} \dot{x}\\ \dot{y}\\ \dot{z}\\ \dot{\psi } \end{array}\right]=\left[\begin{array}{cccc} 0 & 0 & 0 & 0\\ 0 & 0 & 0 & 0\\ 0 & 0 & 0 & 0\\ 0 & 0 & 0 & 0 \end{array}\right]\left[\begin{array}{c} x\\ y\\ z\\ \psi \end{array}\right]+\left[\begin{array}{cccc} 1 & 0 & 0 & 0\\ 0 & 1 & 0 & 0\\ 0 & 0 & 1 & 0\\ 0 & 0 & 0 & 1 \end{array}\right]\left[\begin{array}{c} \dot{x}\\ \dot{y}\\ \dot{z}\\ \dot{\psi } \end{array}\right]$$ (3)

$$\left[\begin{array}{c} x\\ y\\ z\\ \psi \end{array}\right]=\left[\begin{array}{cccc} 1 & 0 & 0 & 0\\ 0 & 1 & 0 & 0\\ 0 & 0 & 1 & 0\\ 0 & 0 & 0 & 1 \end{array}\right]\left[\begin{array}{c} x\\ y\\ z\\ \psi \end{array}\right]+\left[\begin{array}{cccc} 0 & 0 & 0 & 0\\ 0 & 0 & 0 & 0\\ 0 & 0 & 0 & 0\\ 0 & 0 & 0 & 0 \end{array}\right]\left[\begin{array}{c} \dot{x}\\ \dot{y}\\ \dot{z}\\ \dot{\psi } \end{array}\right]$$ (4)

$$\begin{array}{c} \dot{X}=A_{c}X+B_{c}u\\ y=C_{c}X+D_{c}u \end{array}$$ (5)

Continuous to discrete state space conversion equations are shown in Equation 6

Ad=eAcT≈I+AcTBd=eAcττ=0τ=TdτBc≈BcTCd=CcDd=Dc (6)

The converted discrete state space equations can be seen in Equations 7, 8. The short form of these equations can be seen in Equation 9

$${\left[\begin{array}{c} x\\ y\\ z\\ \psi \end{array}\right]}_{t+1}=\left[\begin{array}{cccc} 1 & 0 & 0 & 0\\ 0 & 1 & 0 & 0\\ 0 & 0 & 1 & 0\\ 0 & 0 & 0 & 1 \end{array}\right]{\left[\begin{array}{c} x\\ y\\ z\\ \psi \end{array}\right]}_{t}+\left[\begin{array}{cccc} \delta t & 0 & 0 & 0\\ 0 & \delta t & 0 & 0\\ 0 & 0 & \delta t & 0\\ 0 & 0 & 0 & \delta t \end{array}\right]{\left[\begin{array}{c} \dot{x}\\ \dot{y}\\ \dot{z}\\ \dot{\psi } \end{array}\right]}_{t}$$ (7)

$${\left[\begin{array}{c} x\\ y\\ z\\ \psi \end{array}\right]}_{t}=\left[\begin{array}{cccc} 1 & 0 & 0 & 0\\ 0 & 1 & 0 & 0\\ 0 & 0 & 1 & 0\\ 0 & 0 & 0 & 1 \end{array}\right]{\left[\begin{array}{c} x\\ y\\ z\\ \psi \end{array}\right]}_{t}+\left[\begin{array}{cccc} 0 & 0 & 0 & 0\\ 0 & 0 & 0 & 0\\ 0 & 0 & 0 & 0\\ 0 & 0 & 0 & 0 \end{array}\right]{\left[\begin{array}{c} \dot{x}\\ \dot{y}\\ \dot{z}\\ \dot{\psi } \end{array}\right]}_{t}$$ (8)

$$\begin{array}{c} X_{t+1}=A_{d}X_{t}+B_{d}u_{t}\\ y_{t}=C_{d}X_{t}+D_{d}u_{t} \end{array}$$ (9)

Accurate trajectory tracking benefits greatly from optimal control strategies, which provide advantages over conventional non-optimal methods such as PID controllers. For this reason, the present work employs an optimal control framework. In particular, MPC approach was designed due to its robustness in trajectory tracking and its capability to adjust the vehicle’s motion by anticipating future waypoints.

$$\begin{array}{c} X_{a_{t}}=AX_{a_{t-1}}+Bu_{t-1}\;\\ Y_{a_{t}}=CX_{a_{t}}+Du_{t} \end{array}$$ (10)

Let n be the horizon window of the MPC. The MPC framework seeks to minimize a quadratic cost function, as shown in Equation 11. Let $r_{t}$ be the reference point and $y_{a_{t}}$ be the current state of the UAV, then the error can be expressed as $e_{t}=r_{t}-y_{a_{t}}\;\Rightarrow \;\;e_{t}=r_{t}-C_{d}X_{a_{t}}$ . This state cost function consists of two components Running cost (t = 1 from t = n-1) and terminal cost (t = n). In addition to minimizing the state error, it is also important to minimize the control effort. Excessive control input can result in overly aggressive or unstable behavior. To address this, a control input cost term is incorporated into the objective function. Q and R represents the state and control costs. High state cost tries to reach the target location as soon as possible, on the otherhand Its formulation incorporates penalties on trajectory deviation, control effort, and variations in control inputs. By balancing these terms, the controller adapts dynamically to changes in the position, enabling smooth and reliable trajectory tracking throughout the mission.

$$J=\frac{1}{2}e_{t+n}^{T}\;S\;e_{t+n}+\frac{1}{2}\overset{n-1}{\underset{i=1}{\sum }}\left[e_{t+i}^{T}\;Q\;e_{t+i}\right]+\frac{1}{2}\overset{n-1}{\underset{i=0}{\sum }}\left[u_{t+i}^{T}\;R\;u_{t+i}\right]$$ (11)

In this formulation, $X_{a}$ denotes the UAV state, while $X_{g}$ represents the UGV state. For path following, the UAV is directly assigned predefined waypoints. In contrast, for dynamic trajectory tracking, the UAV’s reference path is generated in real time based on the UGV’s current position, enabling continuous online tracking. The reference generator, expressed in Equation 12, uses the UGV state as input to compute the UAV’s target position.

To maintain consistent altitude, the UAV is commanded to fly at a fixed vertical offset relative to the UGV. The desired altitude $z_{f}$ is set to 8 m above the UGV’s current position, ensuring line-of-sight visibility and stable flight during cooperative operations.

$$\left[\begin{array}{c} x_{r}\\ y_{r}\\ z_{r} \end{array}\right]=\left[\begin{array}{c} x_{g}\\ y_{g}\\ z_{g} \end{array}\right]+\left[\begin{array}{c} 0\\ 0\\ z_{f} \end{array}\right]$$ (12)

### 3.2 Kalman filtering

Kalman filtering was applied exclusively for dynamic trajectory tracking of the UGV. Although the UGV continuously transmits its positional data to the UAV over the network, a Kalman Filter (KF) was implemented on the UAV side to improve robustness and address variations in update frequency. In practice, GNSS data rates may fluctuate due to environmental conditions or communication delays. To maintain smooth and reliable tracking under such circumstances, the KF estimates the UGV’s state, generating a continuous and stable reference trajectory for the UAV to follow.

For state estimation, the UGV’s motion model was linearized and expressed in discrete-time state-space form, as shown in Equation 13. The state vector consists of positional coordinates ${\left[x;y;z\right]}^{T}$ . While the UGV moves on flat terrain, the altitude component z was included to enforce a constant vertical offset (typically 8 m) between the UAV and UGV, thereby accounting for terrain irregularities and ensuring consistent altitude control during tracking. Process and observation uncertainties are represented through the covariance matrix in Equation 14. This formulation encodes the confidence associated with each state variable, enabling the KF to properly weigh measurements and achieve improved filtering and smoothing in real-time operation.

$${\left[\begin{array}{c} x_{g}\\ y_{g}\\ z_{g} \end{array}\right]}_{t}=\left[\begin{array}{ccc} 1 & 0 & 0\\ 0 & 1 & 0\\ 0 & 0 & 1 \end{array}\right]{\left[\begin{array}{c} x_{g}\\ y_{g}\\ z_{g} \end{array}\right]}_{t-1}+\left[\begin{array}{ccc} \delta t & 0 & 0\\ 0 & \delta t & 0\\ 0 & 0 & \delta t \end{array}\right]{\left[\begin{array}{c} \dot{x_{g}}\\ \dot{y_{g}}\\ \dot{z_{g}} \end{array}\right]}_{t-1}$$ (13)

$$P_{t}=\left[\begin{array}{ccc} \sigma_{x_{g}}^{2} & \rho_{x_{g}y_{g}} & \rho_{x_{g}z_{g}}\\ \rho_{x_{g}y_{g}} & \sigma_{y_{g}}^{2} & \rho_{y_{g}z_{g}}\\ \rho_{x_{g}z_{g}} & \rho_{y_{g}z_{g}} & \sigma_{z_{g}}^{2} \end{array}\right]$$ (14)

$\sigma_{x}^{2}$ , $\sigma_{y}^{2}$ , $\sigma_{z}^{2}$ represents the variance. $\rho_{xy}$ , $\rho_{yz}$ , $\rho_{xz}$ represents the covariance. Typically $\rho_{xy}=\rho_{yz}=\rho_{xz}=0$ .

#### 3.2.1 Prediction

During the prediction step, the Kalman Filter estimates the UGV’s future state using its current position and velocity. This step is particularly important when GNSS updates are delayed or briefly unavailable, as it allows the system to propagate the state forward through the motion model and maintain a reasonable estimate of the UGV’s position. The corresponding state prediction equation is provided in Equation 15.

Simultaneously, the state covariance matrix is updated to represent the growing uncertainty over time. As shown in Equation 16, the process noise covariance Q accounts for uncertainties in the UGV’s dynamics and potential external disturbances. Together, these predictive updates enable smoother UAV tracking performance and preserve stability even when GNSS reception is intermittent.

$${X_{G}}_{t}^{-}=A{X_{G}}_{t-1}^{+}+Bu_{t-1}$$ (15)

$$P_{t}^{-}=AP_{t-1}^{+}A^{T}+Q_{t}$$ (16)

#### 3.2.2 Update

The update step of the Kalman Filter is executed whenever new GNSS measurements from the UGV are received. This stage refines the predicted state, improving localization accuracy. Since the UGV employs RTK-enabled GNSS, the associated measurement uncertainty is low, allowing for highly reliable corrections to the estimated state.

The state correction is expressed in Equation 17, where K is the Kalman gain, $X_{G_{t}}$ represents the GNSS-derived position, and H is the observation matrix mapping the predicted state to the measurement domain. The corresponding covariance update is given in Equation 18, representing the confidence in the incoming GNSS data. By incorporating these measurements, the filter reduces estimation error and ensures that the UAV maintains an accurate, real-time estimate of the UGV’s position, thereby supporting reliable coordinated tracking.

$${X_{G}}_{t}^{+}={X_{G}}_{t}^{-}+K_{t}\left({X_{G}}_{t}-H_{t}{X_{G}}_{t}^{-}\right)$$ (17)

$$P_{t}^{+}=\left(I-K_{t}H_{t}\right)P_{t}^{-}$$ (18)

### 3.3 Trajectory generator

To assess the effectiveness of the proposed MPC strategy, two static and one dynamic trajectories were designed: Row-Crop Pattern, Figure-Eight Trajectory and UGV Trajectory.

#### 3.3.1 Row-Crop Pattern

The Row-Crop Pattern was chosen because of its direct relevance to agricultural operations, where ground and aerial vehicles frequently navigate in linear passes between crop rows. This path is generated by alternating X- and Y-axis values across multiple iterations, producing the characteristic back-and-forth motion typical of field coverage tasks. The primary objective of the MPC controller is to track this predefined path with high accuracy, demonstrating its suitability for structured agricultural missions. The row crop pattern generation trajectory is shown in Algorithm 1.

Algorithm 1 Row Crop Pattern Trajectory.

1:  **function** TRAJECTORY_SETUP
2:   Initialize x←0 , y←0 , direction←1

3:   Define $x_{\text{step}}\leftarrow 10$ , $y_{\text{step}}\leftarrow 5$ , $num_{\text{steps}}\leftarrow 5$

4:   Initialize empty lists: traj_x,traj_y,traj_psi

5:   **for** i=1 to $num_{\text{steps}}$ **do**
6: $x_{\text{new}}\leftarrow x+direction\times x_{\text{step}}$

7: $x_{\text{segment}}\leftarrow$ linear space from x to $x_{\text{new}}$ (10 points)
8: $y_{\text{segment}}\leftarrow y$ (same size as $x_{\text{segment}}$ )
9: $\psi_{\text{segment}}\leftarrow 0$ if direction=1 , else π

10:     Append $x_{\text{segment}},y_{\text{segment}},\psi_{\text{segment}}$ to trajectory
11: $x\leftarrow x_{\text{new}}$

12: $y_{\text{new}}\leftarrow y+y_{\text{step}}$

13: $y_{\text{segment}}\leftarrow$ linear space from y to $y_{\text{new}}$ (5 points)
14: $x_{\text{segment}}\leftarrow x$ (same size as $y_{\text{segment}}$ )
15: $\psi_{\text{segment}}\leftarrow \frac{\pi }{2}$ ⊳ Move upward
16:     Append $x_{\text{segment}},y_{\text{segment}},\psi_{\text{segment}}$ to trajectory
17: $y\leftarrow y_{\text{new}}$

18: direction←−direction ⊳ Flip direction for next row
19:   **end for**
20:   Convert traj_x,traj_y,traj_psi to arrays
21:   **return** traj_y,traj_x,traj_psi

22:  **end function**

#### 3.3.2 Figure-eight trajectory

Alongside the Row-Crop Pattern, a Figure-Eight trajectory was developed to evaluate the UAV’s performance under more demanding flight conditions. This path introduces continuous turns and frequent directional changes, serving as a benchmark for assessing the controller’s responsiveness and stability during complex maneuvers. By requiring smooth transitions across curved segments, the Figure-Eight trajectory provides a rigorous test of the MPC framework’s ability to maintain accurate tracking. The eight pattern trajectory generation is shown in Algorithm 2.

Algorithm 2 Figure Eight Trajectory.

1:  **function** Trajectory_Setup_Eight
2:   Define t← linspace from 0 to 2π with 30 points
3:   Set normalize←5

4:   Compute X←sin(t)×normalize

5:   Compute Y←sin(t)⋅cos(t)×normalize

6:   **return** X,Y

7:  **end function**

#### 3.3.3 UGV trajectory

The UGV is configured with a Cube Orange controller with ArduRover firmware that stores a predefined row-crop trajectory. Once initiated through Mission Planner, the vehicle autonomously follows this path without requiring manual control. In practice, the UGV velocity is roughly 1–2 m/s. In parallel, a companion computer executes a script that continuously retrieves state information—including position and velocity, from the flight controller. These data are transmitted over a Wi-Fi link, allowing the UAV to access the UGV’s real-time coordinates for tracking and cooperative navigation. All of this is shown in Algorithm 3.

Algorithm 3 UGV Operation: Autonomous Navigation and State Transmission.

1:  **Input:** Row-crop trajectory uploaded to flight controller
2:  **Output:** Real-time UGV state transmitted to UAV
3:  **Initialization:**
4:  Activate mission via Mission Planner
5:  Begin execution of row-crop trajectory using onboard flight controller
6:  **while** UGV mission is active **do**
7:   **[Companion Computer]**
8:   Read current UGV state (position, velocity) from flight controller
9:   Transmit state data to UAV via Wi-Fi
10:  Wait for next update interval Δt

11: **end while**

## 4 Results

### 4.1 Autonomous takeoff and landing

**FIGURE 5:**
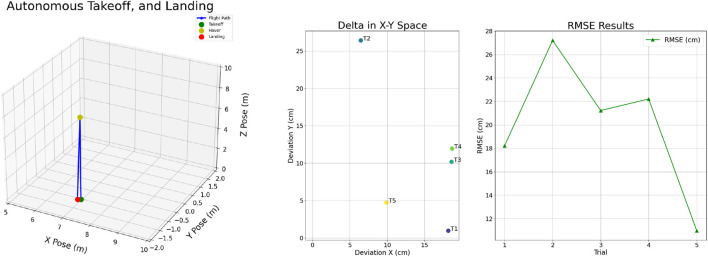
Autonomous takeoff and landing performance showing flight sequence (left) and deviation with RMSE (right).

This sub-section presents the results of the autonomous takeoff and landing trials conducted with our custom UAV. Oftentimes, to perform operations such as spraying and inspection, the vehicle needs to be precisely positioned at a specific point and must be capable of landing on that particular location. Therefore, we conducted this autonomous takeoff and landing test to evaluate how accurately our custom-built UAV can hover and land at a given location. To assess the system’s precision and repeatability, the vehicle was tasked with performing autonomous takeoff and landing maneuvers across five trials. These tests were designed to evaluate the consistency of the UAV’s performance in returning to a designated landing position after each flight. Figure 5 (left) illustrates one of such flights, X-axis illustrates the Front-Back axis, Y-axis shows the Left-Right axis and Z-axis shows the Up-Down axis. The figure shows autonomous takeoff and landing.

The Table 1 summarizes the findings, highlighting deviations along the X-axis (left-right movement) and Y-axis (front-back movement). Figure 5 (right) the left subplot shows the deviation in the UAV’s landing position across five trials in the X-Y plane, where each point (T1–T5) represents the deviation from the intended landing spot along the lateral (X) and longitudinal (Y) axes. This highlights the spatial accuracy of the UAV in autonomous return-to-land operations. The right subplot presents the Root Mean Square Error (RMSE) for each trial, measuring the overall deviation magnitude. For these trials, we excluded values along the Up–Down (vertical) axis, as standard GNSS sensors typically do not provide reliable altitude information at ground level, leading to inconsistencies in the measurement of vertical deviations upon landing. Additionally, we computed the total deviation as the root mean square (RMS) of the deviations along the X and Y-axes. This RMS value provides a comprehensive measure of the overall precision of each landing relative to the designated target. The results indicate that the UAV achieved a maximum deviation of 28 cm and a minimum deviation of 10 cm from its initial location. These findings underscore the UAV’s capability for precise autonomous takeoff and landing, reflecting the effectiveness of the vehicle back to the original landing zone with minimal error.

**TABLE 1:** Autonomous takeoff and landing results.

Test	Deviation X (cm)	Deviation Y (cm)	RMSE (cm)
1	18.19	0.96	18.21
2	6.47	26.42	27.20
3	18.63	10.16	21.22
4	18.71	11.94	22.20
5	9.90	4.72	10.97

### 4.2 Autonomous takeoff, navigation, landing and return to launch

**FIGURE 6:**
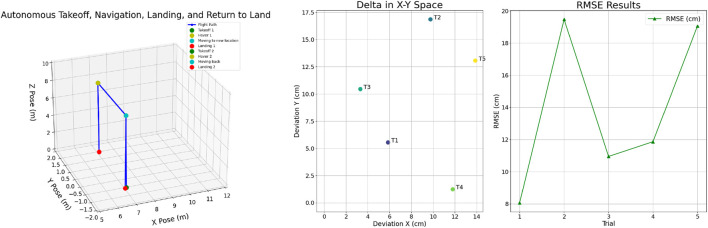
Autonomous takeoff, navigation, landing, and return-to-land performance showing mission sequence (left) and deviation with RMSE (right).

In this scenario, the vehicle was programmed to perform an autonomous takeoff, navigate along either the X or Y-axis, and then land at a predetermined position. Following this landing, the vehicle took off again and autonomously returned to its initial position to perform a final landing. This test was conducted to evaluate the UAV’s capability to execute precise autonomous takeoffs, navigate accurately along designated axes, and return to a specified landing point. Additionally, the vehicle’s ability to reliably return to its original position after completing its mission was assessed. To verify repeatability and robustness, the test was conducted five times. Figure 6 (left) illustrates one of the tests where the vehicle took off, navigate along Y-axis, land and performs return to landing operation. The flight path is shown in blue color, and the scatter point illustrates each phase of the operation.

The results of these trials are presented in Table 2, which includes the deviations observed along the X-axis, Y-axis, and the root mean square error (RMSE) calculated over both axes. The data indicate that, upon returning to the original position after each trial, the UAV was at most 20 cm and at least 8 cm from the initial location. Figure 6 (right) visualizes the deviation in X-Y space for five autonomous flight trials (T1–T5), where the UAV performed takeoff, navigated to a target, landed, and returned to the original location. The scatter points represent the horizontal deviation from the intended return position, showing high repeatability and spatial accuracy across trials. The right subplot illustrates the Root Mean Square Error (RMSE) for each trial, providing a quantitative measure of landing precision. These results highlight the robustness and precision of the UAV’s autonomous navigation and positioning capabilities, demonstrating its effectiveness in maintaining spatial accuracy when returning to its starting point following mission completion.

**TABLE 2:** Autonomous takeoff, navigation and landing results.

Target	Deviation X (cm)	Deviation Y (cm)	RMSE (cm)
1	5.86	5.54	8.07
2	9.76	16.85	19.47
3	3.32	10.44	10.95
4	11.81	1.23	11.87
5	13.88	13.06	19.06

### 4.3 Autonomous row crop pattern tracking

**FIGURE 7:**
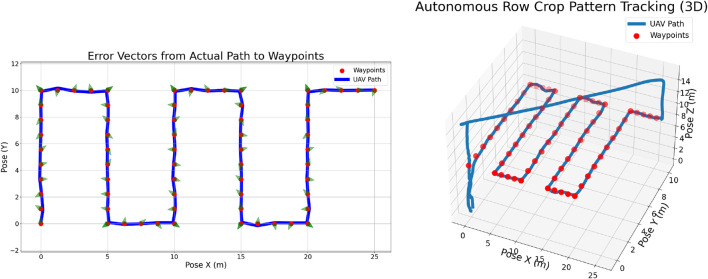
Autonomous row crop pattern tracking showing 2D view (left) and 3D view (right).

This subsection presents MPC performance on the Row-Crop Pattern. Figure 7 (right) illustrates MPC-based tracking of the Row-Crop Pattern. Red dots mark trajectory waypoints, and the UAV’s actual path is shown in blue. The UAV follows the planned trajectory with only minor deviations, attributed to external disturbances or transient control effects, confirming the effectiveness of the MPC approach. The figure also captures the UAV’s autonomous takeoff and return-to-launch (RTL), illustrating the complete end-to-end mission.

Figure 7 (left) depicts the deviation of the UAV’s trajectory from the planned waypoints. The X and Y-axes denote Left–Right and Front–Back directions (in meters). Red dots mark waypoints, the blue line shows the actual path, and green arrows indicate the direction of deviation. This visualization highlights both the accuracy of trajectory tracking and the locations where minor deviations occurred.

To assess the repeatability of the MPC-based trajectory tracking, five experimental trials were conducted. The outcomes of these trials are presented in Table 3, which reports the mean deviations along the X- and Y-axes together with the overall Root Mean Square Error (RMSE). Across all trials, the largest average deviation from the reference path was approximately 16 cm, while the smallest was about 9 cm. These findings highlight the reliability and consistency of the MPC controller in maintaining accurate trajectory tracking.

**TABLE 3:** Autonomous RowCrop pattern navigation results.

Target	Deviation X (cm)	Deviation Y (cm)	RMSE (cm)
1	5.92	5.99	8.42
2	6.58	6.72	9.40
3	12.15	10.33	15.94
4	12.91	14.18	19.18
5	7.42	7.95	10.88

### 4.4 Autonomous eight pattern tracking

This experiment aimed to assess the UAV’s capability to autonomously follow complex trajectories with high precision. For this purpose, a series of waypoints was arranged in a figure-eight pattern, creating a challenging path that demanded continuous changes in direction and coordinated motion across all axes. The UAV was required to track this trajectory autonomously using the implemented control framework.

**FIGURE 8:**
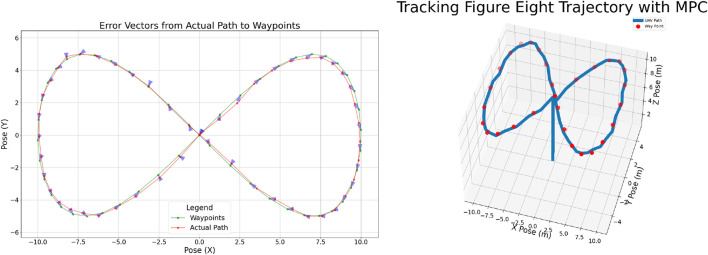
UAV eight pattern trajectory tracking showing 2D trajectory (left) and 3D trajectory (right).

The corresponding results are presented in Figure 8 (right). This figure provides a three-dimensional view of one representative trial, depicting the UAV’s trajectory across the X, Y, and Z-axes, where the red points denote the predefined waypoints and the blue curve illustrates the actual flight path. A comparison of the planned and executed trajectories indicates that the UAV closely adhered to the reference path. These results confirm the system’s ability to manage intricate navigation tasks, demonstrating smooth and accurate path following in real time.

Figure 8 (left) shows the Root Mean Square Error (RMSE) analysis of the UAV’s trajectory relative to the reference waypoints during the figure-eight test. The planned trajectory is shown in green, the actual flight path in red, and blue arrows indicate error vectors pointing from the UAV’s path to the intended waypoints. These vectors highlight both the magnitude and direction of tracking deviations along the trajectory. For most of the path, the error vectors remain short, reflecting accurate tracking. Larger deviations appear near the upper and lower lobes of the figure-eight, where sharper curvature requires rapid control adjustments, increasing the chance of transient errors. This visualization clearly illustrates the spatial distribution of tracking error, demonstrating overall precision while pinpointing the most challenging segments. Such analysis provides useful feedback for refining control strategies in scenarios involving complex maneuvers.

Following the same procedure as in earlier experiments, the figure-eight trajectory was flown five times to assess repeatability and reliability. Table 4 summarizes the results, reporting deviations along the X- and Y-axes together with the Root Mean Square Error (RMSE) derived from these horizontal offsets. The data indicate that the UAV consistently tracked the prescribed figure-eight path with good spatial accuracy. Among the five trials, the largest deviation from the reference waypoints was approximately 35 cm, while the smallest was about 20 cm. These results demonstrate stable performance and the ability of the system to negotiate complex trajectories within moderate error bounds. In summary, the findings confirm the robustness of the autonomous navigation framework in handling curved, non-linear paths. The UAV effectively accommodated the demands of figure-eight maneuvers, reinforcing the validity of the implemented MPC-based control strategy for real-world navigation tasks.

**TABLE 4:** Autonomous eight pattern navigation results.

Target	Deviation X (cm)	Deviation Y (cm)	RMSE (cm)
1	22.78	24.89	33.74
2	12.06	16.03	20.05
3	20.11	23.88	31.22
4	21.98	23.40	32.10
5	21.82	27.34	34.98

### 4.5 Autonomous UAV tracking autonomous UGV

This section reports the results of real-time autonomous tracking, where both the aerial and ground vehicles operated independently. Unlike a simple rectangular route, the UGV traverses a curved row-crop path resembling a figure-eight, which adds complexity due to continuous directional and curvature changes. The UAV effectively adjusted its motion in real time, maintaining successful tracking of the UGV across the full trajectory.

**FIGURE 9:**
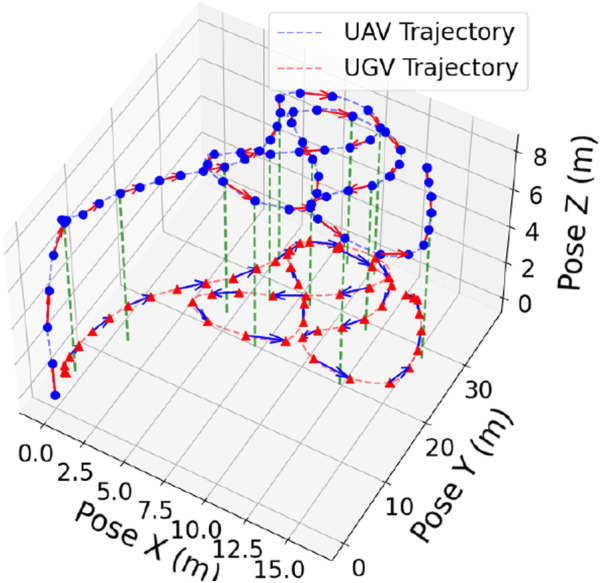
3D trajectories of an autonomous UAV (blue) and UGV (red) with markers at distance intervals. Arrows show motion direction, and green dashed lines indicate UAV altitude relative to ground.

**FIGURE 10:**
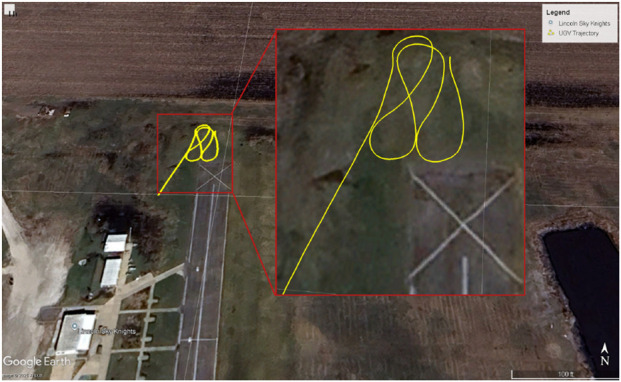
The UGV path at the Lincoln Sky Knights site (40.931627° N, 96.537558° W).

Figure 9 shows the 3D results of the UAV autonomously tracking a UGV. The axes correspond to Front–Back (X), Left–Right (Y), and Up–Down (Z) directions, with all units in meters. In the plot, the UGV’s trajectory is represented in orange and the UAV’s in blue. The UGV follows a predefined row-crop path with figure-eight curvature, introducing sharp turns and frequent directional changes. The UAV continuously adapts its motion in real time to align with the UGV’s updates. The results confirm that the UAV maintained close spatial alignment with the UGV for the duration of the mission, successfully completing the tracking task. Figure 10 illustrates the test field.

### 4.6 Ablation study

The ablation study primarily highlights two aspects: 1. the comparison between simulation and real-world performance, and 2. the effect of prediction horizon length on the MPC controller.

**FIGURE 11:**
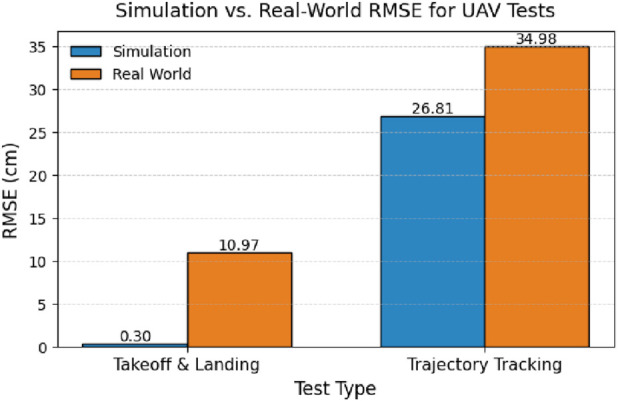
Simulation vs. Real-World RMSE for UAV Tests.

Figure 11 presents the comparison between the simulator and real-world results. It includes the root mean square error (RMSE) values for autonomous takeoff and landing, as well as for figure-eight trajectory tracking. The x-axis represents the test type, and the y-axis denotes the RMSE. As expected, the simulator results exhibit lower errors than those observed in real-world experiments. This discrepancy arises because the simulator operates under idealized conditions, whereas real-world scenarios are influenced by various factors such as wind gusts, sensor noise, actuator imperfections, and environmental disturbances.

**FIGURE 12:**
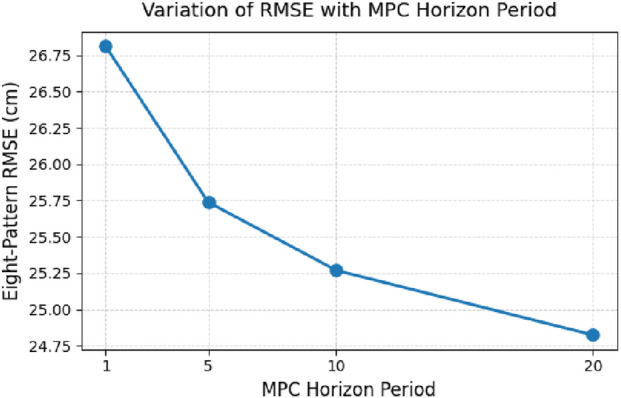
Variation of RMSE with MPC horizon period.

Figure 12 illustrates the impact of varying the horizon period on the MPC controller. The x-axis represents the horizon period length, and the y-axis shows the corresponding RMSE. As the horizon period increases, the RMSE decreases, indicating that a longer prediction horizon enables the controller to anticipate future states more effectively and mitigate errors. However, this improvement comes at the cost of increased computational load. Additionally, a saturation effect can be observed—beyond a certain horizon length, the reduction in RMSE becomes marginal, suggesting diminishing returns from further increases in the horizon period.

## 5 Discussions

### 5.1 Trajectory

The figure-eight pattern was designed and tested with varying numbers of waypoints as well as different lengths and widths of the pattern. The number of waypoints varied between 20 and 50. As the number of waypoints increased, the trajectory became smoother and more closely resembled the figure-eight shape. Conversely, a lower number of waypoints resulted in a trajectory that deviated from the eight-like appearance. Additionally, different lengths and widths ranging from 5 m to 10 m were tested. While all sizes enabled the vehicle to achieve the figure-eight pattern, longer patterns were more visually distinct and pronounced compared to smaller ones.

### 5.2 Control cost

The state cost and control input cost play crucial roles in ensuring smooth trajectory tracking and achieving the goal pose efficiently. A high state cost penalizes errors, ensuring that the vehicle reaches each waypoint as closely as possible. In such cases, the vehicle generates higher velocities to minimize error quickly, resulting in more aggressive behavior. Conversely, a high control input cost constrains velocities, enabling the vehicle to approach waypoints more smoothly, albeit at the cost of increased time to reach each waypoint. The choice between these approaches depends on the mission objective—whether prioritizing rapid target achievement or conserving energy. In our case, we opted for a high control input cost to ensure the vehicle tracks the target smoothly rather than with aggressive maneuvers. Nonetheless, we experimented with both approaches.

### 5.3 Effects of wind

Wind conditions significantly influenced the experimental outcomes. During the testing period, wind speeds ranged between 21 km/h and 29 km/h, with occasional sudden gusts. These wind disturbances caused deviations in the intended figure-eight trajectory, resulting in a distorted or irregular pattern. The effect of wind was more pronounced under the high state cost configuration compared to the high control input cost configuration. In the high state cost scenario, the vehicle prioritized minimizing error by rapidly reaching each waypoint and transitioning to the next. This aggressive approach made the trajectory more prone to deviations, as the vehicle was less capable of compensating for sudden wind gusts, leading to a pattern with noticeable dips and irregularities. Conversely, under the high control input cost configuration, the vehicle moved at a slower pace, taking more time to reach each waypoint. This additional time allowed the vehicle to better adjust to wind disturbances, resulting in a smoother trajectory and greater resilience to gust-induced perturbations.

## 6 Conclusion

This study demonstrated the design, development, and validation of a custom UAV platform tailored for precision agriculture. The system was built with modularity, adaptability, and cost-effectiveness in mind, providing an open alternative to proprietary commercial solutions. A Cube Blue flight controller managed the low-level dynamics, while a Raspberry Pi 4 companion computer executed Model Predictive Control (MPC), enabling efficient trajectory planning and autonomous navigation.

In contrast to conventional autopilot frameworks that rely primarily on PID control and static waypoint missions, the proposed architecture integrates MPC with Kalman filtering to achieve dynamic, real-time tracking of ground vehicles navigating complex, curved paths. The UAV’s capacity to autonomously follow challenging trajectories—such as row-crop and figure-eight patterns—was validated through both simulation (AirSim) and real-world field trials.

Experimental findings confirmed reliable performance, with Root Mean Square Error (RMSE) ranging from 8 to 20 cm in standard takeoff, navigation, and landing tasks, and 20–35 cm for more demanding trajectories. These results underscore the platform’s precision, robustness, and suitability for agricultural operations.

## Data Availability

The original contributions presented in the study are included in the article/supplementary material, further inquiries can be directed to the corresponding author.
